# Recent Insights into the Potential and Challenges of Sericin as a Drug Delivery Platform for Multiple Biomedical Applications

**DOI:** 10.3390/pharmaceutics17060695

**Published:** 2025-05-26

**Authors:** Qisan Ma, Saniya Salathia, Maria Rosa Gigliobianco, Cristina Casadidio, Piera Di Martino, Roberta Censi

**Affiliations:** 1 ChIP Chemistry Interdisciplinary Project Research Centre, School of Pharmacy, University of Camerino, Via Madonna delle Carceri, 62032 Camerino, MC, Italy; 2 Department of Pharmacy, “G. D’Annunzio” of Chieti and Pescara University, Via dei Vestini 1, 66100 Chieti, CH, Italy

**Keywords:** biomaterials, controlled release, nanocarriers, biopolymer engineering, targeted therapy

## Abstract

Sericin, a glycoprotein derived from silk cocoons, has gained significant attention as a versatile biomaterial for drug delivery due to its biocompatibility, biodegradability, and amphipathic nature. This review explores recent advancements in sericin-based drug delivery systems across three key therapeutic domains: antimicrobial applications, anticancer treatments, and neurodegenerative diseases. Various fabrication techniques, including nanoparticles, hydrogels, and microneedles, have been investigated to optimize drug encapsulation, targeted release, and bioavailability. While sericin holds great promise for overcoming challenges associated with synthetic polymers, issues such as molecular variability, formulation stability, and regulatory considerations remain critical hurdles. Future research should focus on optimizing sericin extraction methods, enhancing structural stability, and integrating it with cutting-edge biomedical technologies to maximize its therapeutic efficacy.

## 1. Introduction

The development of effective drug delivery systems is a key aspect of modern therapeutic strategies [1]. The increasing interest in biopolymers for delivery purposes is due to their natural origin, compatibility with biological systems, and ability to be engineered for diverse applications [2]. More recently, biopolymers such as chitosan, alginate, and silk proteins have attracted greater interest because of inherent characteristics that overcome many disadvantages associated with synthetic polymers, including poor biocompatibility, limited biodegradability, and environmental concerns [3,4]. Among these, silk sericin, a glycoprotein derived from the silk cocoon, is emerging as a promising candidate for drug delivery systems [5]. Historically, sericin was largely considered a waste product in the silk industry and was often discarded during the degumming process of silk fibers. However, traditional medicinal practices in East Asia recognized the therapeutic properties of sericin-rich silk water, using it for its reported skin-soothing and anti-inflammatory effects [6]. Early scientific studies in the mid-20th century began to explore its biochemical composition and biological properties, laying the groundwork for its modern biomedical applications. These early insights have since evolved into a more sophisticated understanding of sericin potential, especially in pharmaceutical and regenerative medicine contexts [7]. Its biocompatibility and biodegradability make sericin unique and an environmentally friendly alternative to lower the risk of toxic accumulation in the body [8]. Additionally, sericin exhibits an amphipathic nature, which allows it to interact with both hydrophilic and hydrophobic drugs, enabling the development of versatile delivery platforms [9]. All these features make sericin a promising candidate for addressing the current needs in drug delivery.

In addition to its intrinsic characteristics, sericin also possesses several practical advantages. It is obtainable as a by-product of silk manufacturing, making it economically advantageous and environmentally sustainable [10]. Recent chemical modification routes and biomaterial processing developments have substantially improved its applicability, facilitating the formation of nanoparticles, hydrogels, and composite systems specifically designed for controlled drug release and targeted delivery [11,12,13]. These innovations correspond with a global shift toward personalized and precision medicine, where biopolymers like sericin are part of the formulation of tailor-made treatments for individual patients medical needs.

Despite advances in drug delivery technologies, significant challenges remain in achieving optimal therapeutic outcomes. Conventional delivery systems often suffer from issues such as poor drug stability, insufficient bioavailability, and the lack of a targeted release [1,14,15]. These shortcomings can result in suboptimal drug concentrations at the site of action, increased systemic toxicity, and reduced patient compliance. The design of controlled release systems is particularly challenging. Many synthetic polymers, while effective in some cases, can trigger immune responses, are non-biodegradable, or require complex manufacturing processes [16,17,18]. Moreover, overcoming biological barriers such as the blood/brain barrier (BBB) for neurodegenerative diseases or ensuring selective uptake in cancerous tissues poses additional hurdles [19,20]. Sericin, with its inherent biocompatibility and versatility, has the potential to address these challenges. Its ability to form stable matrices for drug encapsulation enables sustained release, reducing the frequency of drug administration and improving patient compliance [21]. Moreover, the presence of functional groups allows for chemical modifications to increase targeting efficacy [20,22]. These features make sericin a potential candidate in advanced drug delivery systems, especially in fields where the drug pharmacokinetics and pharmacodynamics need to be strictly controlled.

**Figure 1:**
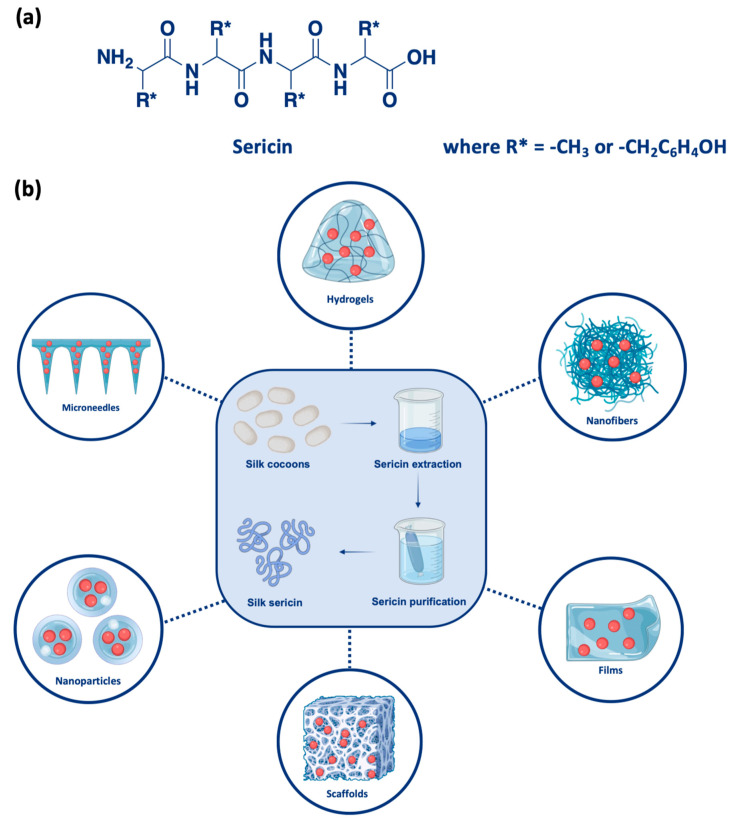
(**a**) Structure of sericin. (**b**) Overview of the fabrication and applications of sericin-based biomaterials. Silk cocoons undergo sericin extraction and purification processes to obtain pure silk sericin, which is further utilized to develop various advanced biomaterials, including hydrogels, nanofibers, films, scaffolds, nanoparticles, and microneedles. These materials are functionalized for diverse biomedical applications such as antimicrobial treatments, anticancer therapies, and neurodegenerative diseases.

**Figure 2:**
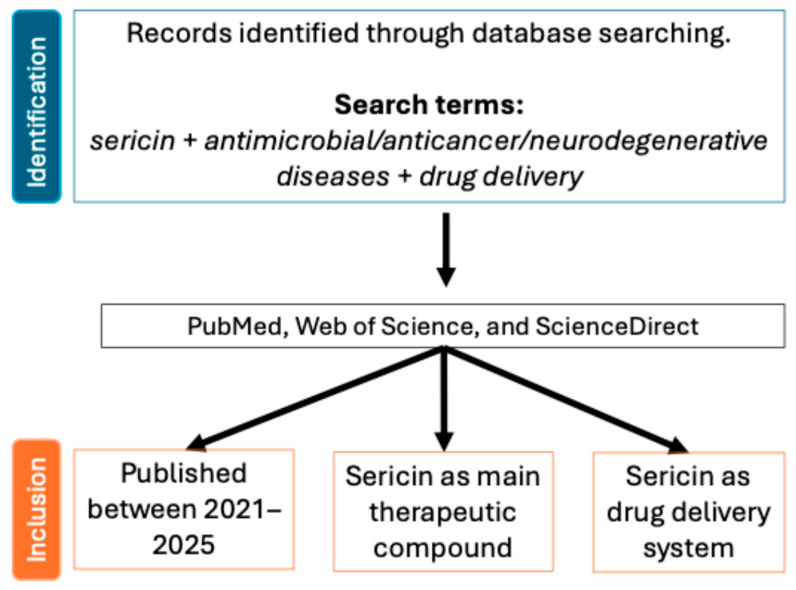
Flow diagram of study selection. Studies published between 2021 and 2025 were included from PubMed, Web of Science, and ScienceDirect, based on sericin being the main therapeutic compound or used as a drug delivery system.

This review aims to explore the recent advancements and future potential of sericin as a polymer for drug delivery systems (Figure 1), with a focus on three critical therapeutic areas: antimicrobial treatments, anticancer therapies, and neurodegenerative diseases. While sericin applications in wound healing are well-documented [23,24], its use in the above-mentioned domains remains relatively underexplored and holds significant promise. By placing the latest advancements from the past four years (2021–2025) in the context of existing research, this manuscript will evaluate the current state of sericin-based drug delivery systems, highlight key challenges, and propose future research directions to address existing gaps (Figure 2). Ultimately, it aims to underscore the remarkable ability of sericin to revolutionize therapeutic treatments and address unmet medical needs.

## 2. Properties of Sericin as a Drug Delivery Polymer

Sericin is an amphipathic protein comprising 18 of the 20 standard (proteinogenic) amino acids, with serine as the predominant component. Its molecular structure is characterized by abundant polar side groups such as hydroxyl (-OH), carboxyl (-COOH), and amino (-NH_2_) groups [25]. These functional groups confer excellent hydrophilicity and reactivity, making sericin well-suited for chemical modifications [26]. Due to variations in amino acid composition, sericin exhibits a broad molecular weight range from 10 to 300 kDa. Its secondary structure is primarily composed of random coils, with a smaller proportion of β-sheet formations [27,28]. The amphipathic nature of sericin derives from its composition of polar and nonpolar amino acids, facilitating the formation of stable emulsions [29]. These properties allow sericin to interact with various drugs through hydrophilic or hydrophobic interactions, making it an excellent candidate for drug delivery applications [30]. Furthermore, the ability of sericin to form hydrogels, films, and nanoparticles via β-sheet induction or covalent crosslinking, upon chemical modification, enhances its potential as a drug carrier for controlled release systems.

Recent research highlights the remarkable biocompatibility and low immunogenicity of sericin, critical for its application in biomedical fields [31,32,33]. Historically perceived as immunogenic, advances in understanding its structure and purification methods have shown that sericin induces minimal immune responses. Specifically, subcutaneous injections of self-cured sericin hydrogels in BALB/c mice showed sericin-specific IgG levels comparable to fibrinogen, an FDA-approved biomaterial [33]. Furthermore, genipin-crosslinked chitosan/sericin hydrogels used as dermal substitutes causing no significant immune response can be found in the work of Sapru et al., where they demonstrated how sericin hydrogels did not induce a notable inflammatory response, as indicated by low TNF-α and IL-1β levels and minimal hemolysis [31]. Additionally, sericin/silk fibroin composites in tissue engineering demonstrated tunable immune properties to enhance biocompatibility [32]. Overall, these findings make sericin particularly suitable for in vivo drug delivery systems.

Sericin also exhibits unique biological activities, including promoting cell adhesion and proliferation, UV protection, and antioxidant properties [34,35]. These inherent activities complement its use as a drug delivery material by supporting tissue regeneration and reducing oxidative stress. Studies demonstrate that hydrazone-bonded pH-sensitive self-assembling doxorubicin/sericin nanoparticles provide a solution to possible drug leaking and provide controlled release structures [36]. Moreover, sericin-based films and hydrogels do not cause significant cytotoxicity, emphasizing their safety in potential clinical applications [37].

The versatility of sericin as a polymer lies in its adaptability to various fabrication techniques, including the development of nanoparticles, hydrogels, and films tailored for specific drug delivery needs (Table 1). Sericin chemical versatility, safety, and adaptability to various fabrication methods underline its potential as a multifunctional drug delivery polymer. The following sections delve deeper into specific therapeutic domains where sericin-based systems have demonstrated significant potential: antimicrobial applications, anticancer drug delivery, and targeted treatments for neurodegenerative disorders.

### 2.1. Sericin Applications in Drug Delivery for Antimicrobial Purposes

Sericin holds innate features essential for antimicrobial applications, including its inherent antibacterial [61,62,63], and antiviral properties [64,65,66], which are attributed to its high content of polar amino acids (e.g., serine and threonine), the presence of reactive functional groups, and its ability to generate reactive oxygen species under UV exposure. Indeed, among natural biomaterials, sericin stands out for its exceptional UV resistance, complementing the higher antibacterial activity of chitosan and antioxidant potency of aloe vera [67]. Beyond antimicrobial uses, sericin supports tissue engineering applications, notably enhancing osteoblast proliferation and reducing biofilm formation by *Staphylococcus aureus*. Its chemical-free extraction and biocompatibility highlight its potential as a sustainable bioactive material for healthcare applications [68].

Research efforts have explored various sericin-based materials to develop innovative solutions addressing microbial resistance and enhancing therapeutic outcomes. One notable application involves the development of a photo-crosslinked bilayer bionic skin scaffold using methacrylated sericin protein (MASF). This scaffold mimics the multilayered structure of human skin, incorporating an antimicrobial outer layer and a pro-angiogenic inner layer [42]. The outer layer, functionalized using tannic acid, demonstrates effective resistance to microbial invasion through its small pore structure and bio-crosslinking properties. Conversely, the inner layer, enriched with dopamine, promotes cell proliferation and supports the wound healing process by maintaining a favorable wound microenvironment. In vitro and in vivo studies confirmed that this bilayer scaffold promotes collagen deposition, supports hair follicle regeneration, and demonstrates excellent mechanical stability, antioxidant properties, and blood compatibility. Importantly, its wound healing performance outperformed the standard commercial wound dressing Tegaderm™ due to its superior regenerative properties [42]. Moreover, sericin-based hydrogels have been developed by incorporating chitosan and resveratrol, forming a multifunctional hydrogel with combined antimicrobial, antioxidant, and anti-inflammatory properties. This hydrogel exhibited excellent spreadability, a porous network structure conducive to cell migration and proliferation, and controlled drug release. These properties facilitated rapid wound closure and reduced bacterial load, which was confirmed through in vivo models. Histopathological analysis further supported the hydrogel ability to promote collagen deposition and accelerate re-epithelialization, demonstrating its potential for wound treatment and therapeutic interventions [41]. In a recent work by Deb et al., silk sericin and polycaprolactone (PCL)-based hydrogels, synthesized at room temperature, exhibited excellent biocompatibility, commendable porosity, and substantial swelling capacity, making them promising for biomedical applications [21]. These hydrogels demonstrate controlled drug release (67% in 130 min with 60.32% encapsulation efficiency), antibacterial properties, and biodegradability, highlighting their potential in therapeutic agent delivery.

**Figure 3:**
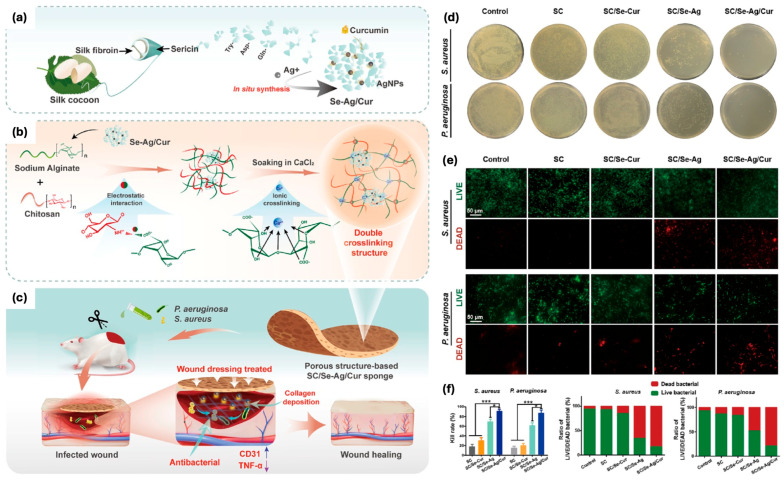
(**a**) Illustration of the sericin−AgNPs/curcumin (SC/Se−Ag/Cur) nanocomposite preparation process. (**b**) Illustration of the SC/Se−Ag/Cur sponge fabrication (**c**) and its use in enhancing infected wound healing. (**d**) Images of *P. aeruginosa* and *S. aureus* colonies treated with SC, SC/Se−Ag, SC/Se−Cur, and SC/Se−Ag/Cur sponges. (**e**) LIVE/DEAD bacterial staining. (**f**) Bacterial kill rates for *P. aeruginosa* and *S. aureus* treated with different sponges, with statistical significance (* *p* < 0.05, *** *p* < 0.001). Adapted with permission from [59].

Sericin has also been utilized in the synthesis of silver nanoparticles (sericin-AgNPs), which were combined with curcumin to create a hybrid antimicrobial agent (Figure 3) [59]. In the work published by Jiang et al., this composite was incorporated into a 3D physically double-crosslinked sponge scaffold made of sodium alginate and chitosan networks. The first layer of the sponge was formed by electrostatic interactions between sodium alginate and chitosan, whereas for the second layer, alginate was ionically crosslinked with calcium ions. The sponge demonstrated excellent properties such as high porosity, good moisture retention, and mechanical strength. Furthermore, in vivo studies revealed that this composite sponge promoted the regeneration of epithelial tissues, reduced inflammation by downregulating the pro-inflammatory cytokine TNF-α, and stimulated angiogenesis by enhancing CD31 expression. These findings position sericin-AgNPs/curcumin scaffolds as effective therapeutic materials for treating bacterial infections and promoting wound healing in infected wounds [59].

Additionally, sericin-AgNPs have been synthesized as eco-friendly antimicrobial agents, exhibiting excellent antibacterial and antifungal activities against a range of bacterial and fungal pathogens. These nanoparticles showed stability across different temperature ranges, supporting their effectiveness under diverse environmental conditions. The broad-spectrum activity of sericin-AgNPs, combined with their biocompatibility and green synthesis process, positions them as promising agents for wound treatment and antimicrobial drug delivery systems [38]. Additionally, scaffolds derived from eri silk, produced by domesticated silkworms, have emerged as a promising biocompatible material for antimicrobial wound treatments. Silver ions were reduced, when mixed with sericin, to form AgNPs-coated sericin scaffolds. These scaffolds, decorated with AgNPs via a natural reduction process facilitated by sericin and fibroin, demonstrated excellent antibacterial efficacy against multidrug-resistant pathogens such as *Staphylococcus aureus* and *Pseudomonas aeruginosa*. As compared to more common mulberry silk, eri silk scaffolds showed superior structural, mechanical, and thermal stability. The increased hydrophobicity made them optimal for standard waterproof dressing material. Hence, eri silk scaffolds make a good option for burn wound treatments and other chronic wound management strategies [51]. Another study carried out by Shaw et al. demonstrated the role of silk sericin as a reducing and stabilizing agent in synthesizing AgNPs for antibacterial and food preservation applications. The sericin-AgNPs effectively inhibited spoilage bacteria and, when used as a food coating, extended the shelf life of tomatoes by reducing microbial contamination. With confirmed biocompatibility and antimicrobial efficacy, this research highlights sericin’s potential to transform waste into valuable resources for sustainable nanotechnology applications in food packaging and biomedical fields [40].

Furthermore, sericin/alginate/aloe vera scaffolds have emerged as effective multifunctional wound dressings. These scaffolds exhibited superior hemostatic, antimicrobial, and angiogenic properties and demonstrated strong compatibility with blood and tissue. This multifunctionality positions sericin/alginate/aloe vera scaffolds as ideal candidates for emergency hemostasis and advanced wound healing strategies [45].

Sericin-coated electrospun nanofibers represent another promising application of sericin-based drug delivery systems. AgNPs-functionalized sericin was successfully adsorbed onto the surface of nanofibers by Gök et al., demonstrating excellent antimicrobial properties. These nanofiber membranes exhibited significant antibacterial activity against both gram-positive and gram-negative bacteria, such as *E. coli* and *S. aureus*. When evaluated in burn wound healing models, these sericin-nanofiber scaffolds outperformed commercial dressings, supporting faster wound closure and improved tissue regeneration. Additionally, cytotoxicity assays confirmed the biocompatibility of these scaffolds, validating their safety for biomedical applications [54].

**Figure 4:**
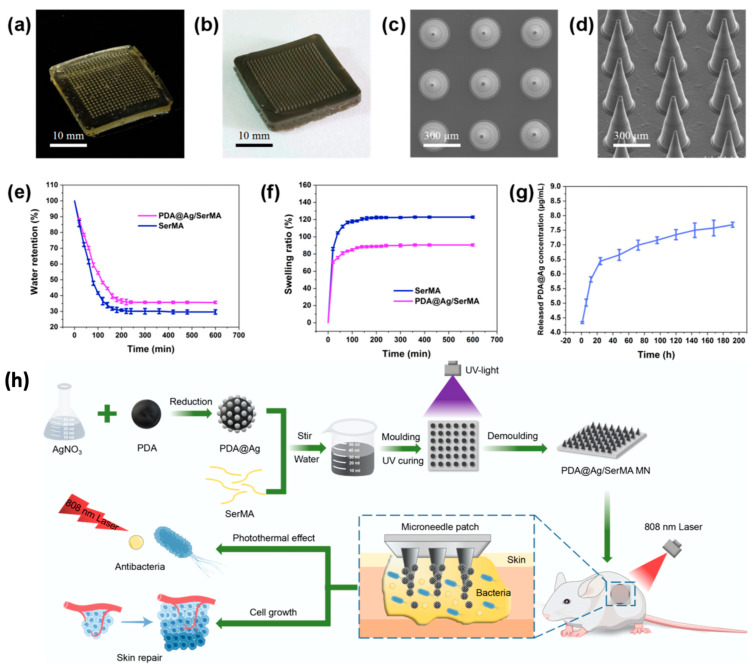
Overview of PDA@Ag/SerMA microneedles. (**a**,**b**) Optical images of SerMA and PDA@Ag/SerMA microneedles. (**c**,**d**) SEM images of SerMA microneedles. (**e**,**f**) Water retention and swelling ratio curves for SerMA microneedles before and after loading PDA@Ag NPs (n = 3). (**g**) Ag^+^ release curve from PDA@Ag/SerMA microneedles (n = 3). (**h**) Schematic of the preparation and application of the PDA@Ag/SerMA microneedle patch, which enhances wound healing through sustained Ag^+^ release and photothermal antimicrobial effects. Adapted with permission from Chen et al. Copyright (2024) American Chemical Society [48].

Innovative microneedle platforms have also incorporated sericin derivatives for advanced drug delivery applications. In particular, PDA@Ag/SerMA microneedles (Figure 4) were developed by combining the photothermal properties of polydopamine (PDA), the antimicrobial properties of silver (Ag), and the regenerative properties of sericin methacryloyl (SerMA). Photothermal therapy damages the structures of bacteria, which makes them less resistant to antimicrobials. The additional heat generated from photothermal therapy triggers the release of antimicrobial agents from the photothermal material. The photothermal property of PDA is enhanced when it is functionalized with Ag and loaded in a hydrogel. SerMA is a modified form of sericin that can be UV-crosslinked into hydrogel-forming microneedles and provide a carrier for Ag-modified PDA. Under near-infrared (NIR) laser irradiation, these microneedles achieved near-complete bacterial elimination against *S. aureus* and *E. coli*, while also facilitating wound healing. This combination of photothermal, antimicrobial, and regenerative functionalities offers a minimally invasive and effective treatment for diabetic foot ulcers and other chronic wound types [48].

Further advancements include the development of nanofiber drug delivery systems using sericin as a matrix for controlled drug delivery. These systems incorporated halloysite nanotubes (HNTs) loaded with chlorhexidine acetate (CA), a well-known antimicrobial agent [53]. The HNTs were mixed with a sericin-based polyurethane (PU/SS) solution and electrospun to design nanofibers for wound dressing. The PU/SS nanofibers exhibited controlled drug release kinetics with a 25% release after 7 days, as compared to a 35% release from CA-HNTs with no PU/SS. The double-layer drug-loaded system with HNTs and PU/SS lead to a reduced initial burst effect commonly associated with conventional drug delivery methods, and a higher storage stability. The sustained release mechanism provided prolonged antimicrobial action against *S. aureus* and *E. coli*, proving effective for chronic wound treatment and sustained therapeutic delivery. Cytocompatibility studies demonstrated that sericin promotes cell proliferation, further supporting its role as a safe and efficient drug delivery vehicle [53].

Finally, sericin/agarose composite films, combined with zinc oxide (ZnO) and AgNPs via layer-by-layer self-assembly, demonstrated significant antimicrobial efficacy. These composite films exhibited excellent mechanical properties, hydrophilicity, and antibacterial action against both gram-positive and gram-negative bacteria, making them suitable for wound dressings, antibacterial coatings, and advanced tissue engineering applications [46].

Sericin-based platforms provide versatile and innovative solutions for drug delivery and antimicrobial applications. It has proved its versatility to be used as nanofibers, films and microneedles. However, more research with different sources of sericin may open avenues in improved mechanical and structural strength.

### 2.2. Sericin Applications in Drug Delivery for Anticancer Purposes

In addition to antibacterial properties, silk sericin has shown remarkable versatility in drug delivery systems targeting cancer. The utility of sericin extends beyond conventional applications, as it has demonstrated the ability to protect healthy biological tissues from oxidative damage and cellular apoptosis, which is especially relevant in oncology and regenerative medicine [69,70]. For instance, sericin exhibited significant hepatoprotective effects against diethylnitrosamine (DEN)-induced hepatic injury by reversing pro-inflammatory cytokine levels and mitigating liver structure deterioration, suggesting its role as a promising adjunct in reducing liver toxicity during cancer treatments [71,72]. Moreover, in a dose-dependent manner, sericin acts in the G0/G1 phase to arrest the cell cycle and promote apoptosis in triple-negative breast cancer (TNBC) models. It also suppresses the PI3K/Akt signaling pathway, leading to reduced proliferation and enhanced cellular apoptosis in MDA-MB-468 cells. The antitumor effect of sericin stems from its ability to increase the low expression levels of p21 in the nucleus, which causes DNA damage. This mechanism highlights its potential as a natural therapeutic agent in addressing aggressive cancer subtypes [73]. Sericin multifunctionality is further supported by its ability to activate the extrinsic apoptotic pathway in HCT116 colorectal cancer cells, where it upregulated key caspase-related genes and death receptor pathways. At high concentrations (100 µg/mL), sericin was able to increase the expression levels of multiple apoptotic proteins like p53, BAD, and AKT. This result was accompanied by significant biochemical changes, such as DNA and lipid modifications, underscoring its ability to induce programmed cell death in a controlled manner [74]. The expression levels of these proteins are relatively higher in tumor-cell nuclei as compared to healthy-cell nuclei. Treating tumor cells with sericin increases the expression levels of these proteins to a severe effect.

Recent studies have demonstrated the potential of sericin-based nanoparticles, hydrogels, and films in enhancing the efficacy of anticancer drugs, enabling targeted delivery, and reducing systemic side effects. For instance, sericin/silver nanoparticles (sericin-AgNO3 NPs), synthesized using a degumming process, exhibited significant cytotoxic effects against breast cancer cell lines, MCF-7 and MDA-MB-231, by inducing cell cycle arrest and enhancing the expression of stress-related genes such as CDKN1A and GADD45. Sericin-AgNO3 NPs can increase ROS levels to excessive amounts, which is also the root mechanism of their antibacterial activity. Mumtaz et al. have managed to harness this property of sericin-AgNO3 NPs to act against tumor cells and cause cell disruption and DNA damage. To contain the ROS damage, sericin is used as a hydrophilic polymeric stabilizer and target the effect to tumor cells only. At a concentration of 1 mg/mL, these nanoparticles facilitated nuclear fragmentation and apoptosis, demonstrating their tumor-specific cytotoxicity. However, their potential in clinical settings necessitates further in vivo validation [75]. Another study, conducted by Liu et al., highlighted the development of sericin microparticles, developed using a single-emulsion method, enveloped with metal/organic networks (MONs) for pulmonary drug delivery. These particles, loaded with doxorubicin, demonstrated sustained release, improved drug retention in lung tissues, and a significant inhibition of metastatic nodules in a breast cancer model, showcasing their promise for pulmonary administration [76]. Sericin’s versatility is further illustrated through its conjugation with magnesium oxide nanoparticles (SS-MgO-NPs), which demonstrated a range of therapeutic effects including antibacterial, antioxidant, anti-aging, and anticancer activities. With an IC50 of 207.6 µg/mL against MCF-7 cells, SS-MgO-NPs also inhibited enzymes such as collagenase and tyrosinase, suggesting their application in both pharmaceutical and cosmetic industries [77]. In another innovative approach adopting the solvothermal method, sericin was combined with superparamagnetic iron oxide nanoparticles (SPIONs) for the delivery of siRNA targeting ROR1, a gene overexpressed in triple-negative breast cancer cells. The magnetically guided delivery system effectively silenced the target gene, induced tumor necrosis, and demonstrated its potential as a precision therapeutic tool [58]. Similarly, the nanoprecipitation method of zein/sericin nanoparticles encapsulating 5-fluorouracil (5-FU) preserved the drug pharmacological activity, enabling controlled release and enhanced stability, offering a cost-effective solution for systemic anticancer therapy [55].

The integration of stimuli-responsive properties into sericin-based delivery systems has further advanced their therapeutic potential. For instance, a pH-responsive sericin/zeolitic imidazolate framework loaded with doxorubicin (ZIF-8@DOX@SS) enhanced the stability and tumor-specific release of the drug in acidic environments, significantly improving therapeutic outcomes while minimizing systemic toxicity. Blood hemolysis experiments conducted to analyze biocompatibility showed less than 5% toxicity of all ZIF-8@DOX@SS concentrations (1/5/25/50/100/200 µg/mL) as compared to a positive control. At a low pH, ZIF-8@DOX@SS showed a much more rapid internalization compared to free DOX. This fast cell uptake, combined with a 92% drug release in 2 h, proved ZIF-8@DOX@SS to be the optimal drug carrier in a low pH tumor environment [39]. Similarly, a vitamin B12-sericin-poly(γ-benzyl-L-glutamate)-IR780 (VB12-sericin-PBLG-IR780) complex enabled photodynamic and photothermal therapies (PDT/PTT) in gastric cancer. The encapsulation of IR780 in the VB12-sericin-PBLG complex increased its photostability from 60% to 90% after 12 h of storage and significantly improved its biocompatibility. Upon NIR irradiation, this system targeted mitochondria, induced oxidative stress, and activated antitumor immunity. No damage was reported on cells treated with VB12-sericin-PBLG-IR780 but not irradiated with NIR. When tested in vivo, an 87% decrease in tumor growth was reported in NIR-irradiated VB12-sericin-PBLG-IR780 mice, highlighting sericin’s potential in multifunctional cancer therapy [49].

Sericin’s role in theranostic applications is exemplified by its use in layered double hydroxides (LDHs) modified with ZnO quantum dots and loaded with pemetrexed, as demonstrated by Abdelgalil et al. [57]. These systems, formulated with the novel desolvation technique, combined diagnostic imaging with targeted drug delivery, achieving enhanced uptake and cytotoxicity in breast cancer cells [57]. Furthermore, a sericin/propolis/fluorouracil nanoformulation was shown to inhibit colorectal cancer progression through PI3K/AKT/mTOR pathway modulation, promoting apoptosis and autophagy [47].

The development of smart nanocarriers utilizing sericin has been particularly impactful. The nanoprecipitation method of formulating charge-reversal sericin nanoparticles, designed to co-deliver resveratrol and melatonin, effectively exploited the acidic tumor microenvironment to enhance cellular uptake and induce apoptosis. These nanoparticles demonstrated significant cytotoxicity against breast cancer cells, providing a synergistic therapeutic effect [56]. Similarly, chlorin e6-conjugated self-assembling sericin nanoparticles (SSC NPs) offered enhanced photodynamic therapy by improving tumor cell uptake by 94% compared to the control and a 30% increase in cytotoxicity upon irradiation. In vivo studies in tumor bearing mice models showed the retention of SSC NPs only at the tumor sites, due to the enhanced permeability and retention (EPR) effect, leading to minimal off-target effects [78].

Facing the multiple and challenging characteristics of tumors, chemodynamic therapy (CDT) is an emerging therapeutic approach. It uses toxic hydroxyl radicals (OH·) produced by the Fenton reaction to induce cell apoptosis, which is not restricted by other factors such as the limitations of tissue depth and hypoxic tumor microenvironment. However, the problem of insufficient endogenous hydrogen peroxide (H_2_O_2_) seriously restricts its development and widespread use. In the study conducted by Hou et al. [79], a new type of nano-Fenton catalyst SS-CuO_2_@Dox (SCD) was developed, prepared by the peroxidation reaction of Cu^2+^. On being encapsulated in sericin, it exhibited excellent hydrophilicity and biocompatibility, targeting tumor regions via the EPR effect. It decomposed under the acidic conditions of the tumor microenvironment (TME), endogenously releasing H_2_O_2_ and Cu^2+^, which enhanced the efficiency of the Fenton reaction. Simultaneously, the delivery of doxorubicin (Dox), a chemotherapeutic drug, realized the combination of chemotherapy and CDT, improving the therapeutic effect on tumors. This strategy of generating H_2_O_2_ in situ could solve the problem of insufficient H_2_O_2_ in tumors without causing damage to other tissues and organs. The results of in vitro and in vivo experiments showed that acidic environments caused SCD NPs to degrade and greatly increase oxidative stress at the tumor site and caused damage to cellular proteins and DNA, leading to apoptosis and the significant inhibition of tumor growth. This study provides new insights for enhanced CDT and synergistic therapies with high efficacy [79].

Rising cases of non-melanoma skin carcinoma (NMSC) and increasing levels of ultraviolet radiations underline a significant correlation with environmental deterioration and increasing health issues. Current therapeutics have not effectively controlled recurrence rates. The work of Nayak et al. [50] initiated a cost-effective and biodegradable therapy by exploring four formulations combining AgNPs, sericin (isolated from cocoons of *Antherea mylitta*), and chitosan. Various ethosomal formulations were evaluated as platforms for transdermal delivery vehicles for efficient skin intervention therapeutics. The combination of AgNPs and sericin effectively combated the morphological and cellular deformation of epidermoid A431 skin carcinoma cells by overproducing superoxide (O2) and nitric oxide (NO) radicals, depolarizing the mitochondrial membrane potential, and triggering apoptosis and necrosis. In vivo experiments exhibited the stimulation of IgM secretion with T-cell-mediated immune responses. This study proposes a novel approach for treating NMSC using biocompatible formulations delivered through ethosomes. Engineered ethosomal formulations demonstrated strong potential to replace outdated therapies for NMSC with minimal chances of drug resistance and recurrence, favoring natural, cost-effective resources to enhance therapeutic outcomes with a minimal bio-burden to the environment [50].

Moving towards gene therapy, small interfering RNA (siRNA) is a promising therapeutic modality. While several safe and effective delivery systems such as lipid nanoparticles and lipid complexes have already enabled the commercialization of siRNA-based therapeutics [80], there remains a significant opportunity to develop alternative delivery platforms that can further enhance their clinical performance, targeting efficiency, and broader applicability. SPIONs-based drug or gene carrier systems display tremendous promise in nanomedicine due to their unique superparamagnetism, allowing them to be concentrated at targeted therapeutic sites under an external magnetic field [81]. To overcome challenges such as colloidal instability, reactive oxygen species (ROS) generation, and associated toxicological concerns, a novel sericin-coated SPION system modified with PLL was developed as a siRNA carrier [81]. The resulting PLL/Ser-SPIONs were synthesized using a cost-effective co-precipitation method, and exhibited a favorable size, magnetic properties, high siRNA binding capacity, and low toxicity. Kara et al. demonstrated sustained siRNA release kinetics and high biocompatibility. This system represents a promising siRNA carrier candidate, with future work focusing on investigating specific gene silencing, therapeutic RNA delivery, and MRI imaging for cancer therapy and diagnosis [81].

**Figure 5:**
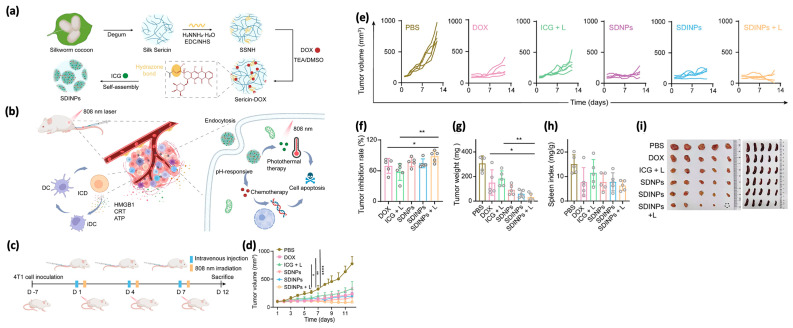
Overview of the pH-responsive sericin nanoplatform for combined photothermal therapy (PTT) and chemotherapy. (**a**) Diagram showing the preparation of the sericin-based nanoplatform, designed to carry the chemotherapeutic drug doxorubicin and the photosensitizer indocyanine green. (**b**) Illustration of the immunogenic cell death (ICD) triggered by the combined therapy. (**c**) Schematic of the treatment process and in vivo antitumor evaluation. (**d**,**e**) Tumor growth curves (average and individual) for all groups (n = 5). (**f**–**h**) Tumor inhibition rates, weights, and spleen indexes measured on day 12 (* *p* < 0.05, ** *p* < 0.01, **** *p* < 0.0001). (**i**) Optical images of tumors and spleens extracted post-treatment. Adapted with permission from [82].

Inducing immunogenic cell death (ICD) by external-stimuli therapy holds great promise for cancer immunotherapy, though challenges such as oxygen depletion limit the efficacy of widely used photodynamic therapy (PDT) and sonodynamic therapy (SDT). Hydrazide-conjugated natural sericin nanoparticles (SDINPs) have been developed as ICD inducers based on photothermal therapy (PTT) to alleviate the immunosuppressive tumor microenvironment (Figure 5). These sericin-based ICD inducers comprise chemically conjugated pH-responsive chemotherapeutic drugs like doxorubicin (DOX) and physically loaded photosensitizers such as indocyanine green (ICG). Their therapeutic ability can be specifically activated by non-invasive laser radiation. Compared to free molecules, nanostructured SDINPs exhibit superior photothermal conversion efficiency, prolonged blood circulation time, and enhanced tumor accumulation, retention, and penetration capabilities. This results in potent therapeutic efficacy and PTT-mediated ICD burst induction for antitumor immunity. This strategy overcomes the challenge of costly raw materials and presents promising prospects for future combination therapies [82].

Furthermore, sericin has shown potential in colorectal cancer therapy, particularly when combined with poly(2-(dimethylamino)ethyl methacrylate) (PDMAEMA) through free radical polymerization. Hybrid nanoparticles loaded with 5-fluorouracil (5-FU) and oxaliplatin (OXP) were developed for targeted colorectal cancer (CRC) therapy. These nanoparticles exhibited excellent biocompatibility and reduced cell viability in HT-29 cells, demonstrating their promise for drug protection and minimized systemic toxicity [83]. Another study focused on the development of sericin-based nanocarriers using flash-nanoprecipitation for cisplatin delivery. These sericin nanoparticles, modified for charge reversal, were designed to optimize controlled drug release and increase drug encapsulation efficiency. When used for cisplatin delivery in breast cancer cells, they exhibited significant cytotoxicity and apoptosis induction, highlighting their potential as a promising nanocarrier for cancer therapy [84].

Sericin antioxidant and anticancer properties have also been investigated for their ability to mitigate toxicity induced by polycyclic aromatic hydrocarbons (PAHs), such as 7,12-dimethylbenzeneanthracene (DMBA), which are known for causing cancer and oxidative stress. Sericin conjugated with silver nanoparticles demonstrated a significant reduction in DMBA-induced toxicity, improving blood parameters, biochemical markers, and histopathological outcomes in a murine model, suggesting its therapeutic efficacy in cancer-related toxicity [85]. Lastly, sericin nanoparticles, made with the nanoprecipitation method and loaded with doxorubicin, were investigated for breast cancer therapy. These nanoparticles, ranging from 25 to 40 nm in size, showed improved drug delivery, enhanced cancer cell DNA damage, and significant reduction in cell viability, emphasizing sericin’s potential as an effective nanocarrier in cancer treatment [86]. Another approach to treat breast cancer involves the use of silk sericin as a protein-based charge-reversal nanocarrier for targeted drug delivery. The direct dissolution method was used to coat sericin on magnetic silica nanoparticles (MSN-PTA-SER). These nanoparticles exhibited superior stability, smaller size, and enhanced negative zeta potential, making them an effective and promising system for improving drug delivery efficiency to breast cancer cells [87].

**Figure 6:**
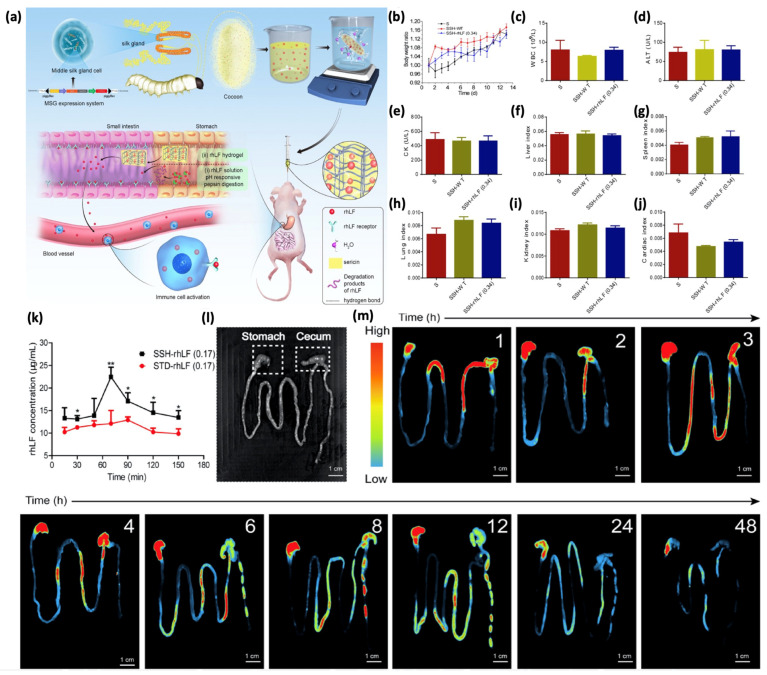
Pharmacokinetic characteristics and in vivo biosafety test of hydrogels. (**a**) Fabrication of the rhLF Sericin hydrogel for improving immunity of immunosuppressed mice. (**b**) Body weight growth of mice after oral administration of SSH-WT and SSH-rhLF (0.34). (**c**) Complete blood count of white blood cells (WBCs). (**d**) Level of glutamic-pyruvic transaminase (ALT) and (**e**) CK that are related to liver and kidney functions. Determination of main organ indices in mice including (**f**) the liver index, (**g**) spleen index, (**h**) lung index, (**i**) kidney index, and (**j**) cardiac index. (**k**) Pharmacokinetic profiles of rhLF in mice that were orally administered with STD-rhLF (0.17) solution or SSH-rhLF (0.17) hydrogels. (* *p* < 0.05; ** *p* < 0.01). (**l**) Bright-field images of the gastrointestinal tract (GIT) from the control group (without rhLF hydrogel treatment). (**m**) Biodistribution of rhLF hydrogels: the mice received oral administrations of rhLF hydrogels, and the fluorescence images of the GIT were obtained at various time points (1, 2, 3, 4, 6, 8, 12, 24, and 48 h). Adapted with permission from Xu et al. Copyright (2021) American Chemical Society [43].

Sericin cancer applications are not limited to therapeutics delivery via nano-systems but also hydrogels and films play a crucial role in mitigating chemotherapy-induced side effects. A silk sericin hydrogel loaded with recombinant human lactoferrin (rhLF) was shown to enhance immune function in cyclophosphamide-treated mice by protecting the spleen and thymus while promoting lymphocyte proliferation (Figure 6). This system offers a novel approach to improving chemotherapy tolerance and efficacy [43]. Another innovative application involved a methacrylic anhydride-modified sericin-based hydrogel dressing for melanoma resection sites, which combined immunogenic cell death induction with infection control, thereby preventing tumor recurrence and promoting wound healing [88]. In the study published by Zhang et al., sericin, combined with temperature-sensitive chitosan, served as a hydrogel delivery system to enhance breast cancer treatment by enabling a precise intratumoral administration and the sustained, on-demand drug release of ROS-sensitive tegafur-protoporphyrin IX (TTP) heterodimers. This approach synergizes chemotherapy and photodynamic therapy, reducing systemic toxicity, overcoming biological barriers, and significantly improving antitumor efficacy under laser irradiation [44].

Additionally, sericin, along with pectin, is utilized as a key component in the formulation of films. It plays a crucial role as a delivery system, enabling the controlled release of simvastatin with enhanced mechanical strength through integration with silver nanoparticles. These films exhibit significant anti-inflammatory and antiangiogenic properties, demonstrated by downregulation of TNF-α and IL-1β in inflammatory models and disruption of blood vessel networks in CAM assays, highlighting their potential in biomedical applications [89].

Overall, sericin adaptability as a biopolymer highlights its broad applicability in anticancer drug delivery. By enabling targeted therapy, reducing adverse effects, and integrating diagnostic capabilities, sericin-based systems align with the goals of precision oncology. However, further research and clinical trials are needed to fully translate these findings into viable treatments. The potential of sericin in personalized medicine is broad and promising, paving the way for transformative advancements in cancer therapy.

### 2.3. Sericin in Drug Delivery for Neurodegenerative Diseases

**Figure 7:**
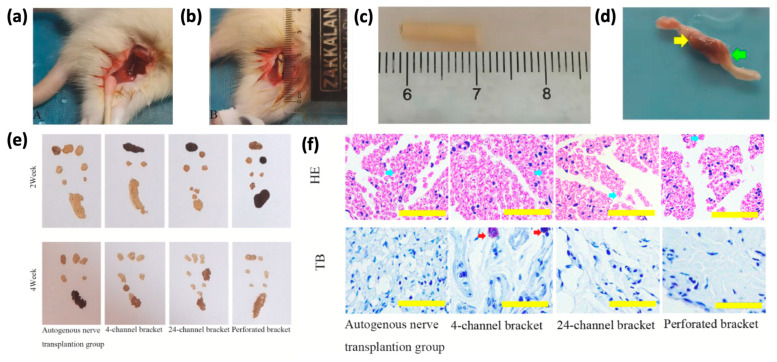
(**a**) Surgical diagram of the autogenous nerve graft group; (**b**) surgical diagram of the stent group; (**c**) photo of the stent before implantation; (**d**) photo of the stent 4 weeks after implantation, with yellow and green arrows showing the proximal and distal ends, respectively. (**e**) Rat footprints from both groups at 2 and 4 weeks. (**f**) HE and TB staining of regenerated nerves 2 weeks post-surgery (scale: 50 μm), with blue arrows marking myelinated axons and red arrows marking neuronal cell bodies. Adapted with permission from [60].

Addressing one of the most significant challenges in treating central nervous system (CNS) diseases, sericin-based drug delivery systems have demonstrated promising potential in overcoming the restrictive nature of the blood/brain barrier (BBB). Although the BBB’s selective permeability serves to protect the brain, it also limits the delivery of therapeutic agents to target areas [90]. Sericin, with its inherent neuroprotective properties [91,92,93], enhances the efficacy of drug delivery systems by encapsulating bioactive compounds, thereby ensuring sustained therapeutic levels and shielding these agents from enzymatic degradation. Advances in biomaterial design have highlighted sericin’s utility in engineering scaffolds tailored for neurological diseases, positioning it as a versatile and promising candidate in CNS therapeutics [94,95]. Recently, developments in biomaterial design have underlined sericin’s role in the production of engineered scaffolds optimized for neural tissue repair. One of the important developments is the preparation of multi-channel scaffolds using silk fibroin and sericin at a ratio of 1:0.5 (Figure 7). This balance ensures optimum porosity, mechanical stability, swelling behavior, and degradation rates. These scaffolds have shown excellent in vivo performance and have promoted Schwann cell adhesion, proliferation, and differentiation, all key to peripheral nerve regeneration. More specifically, four-channel scaffolds have been used to successfully repair 10 mm sciatic nerve defects in rat models with better outcomes in axon regrowth and functional recovery than simpler designs [60].

**Figure 8:**
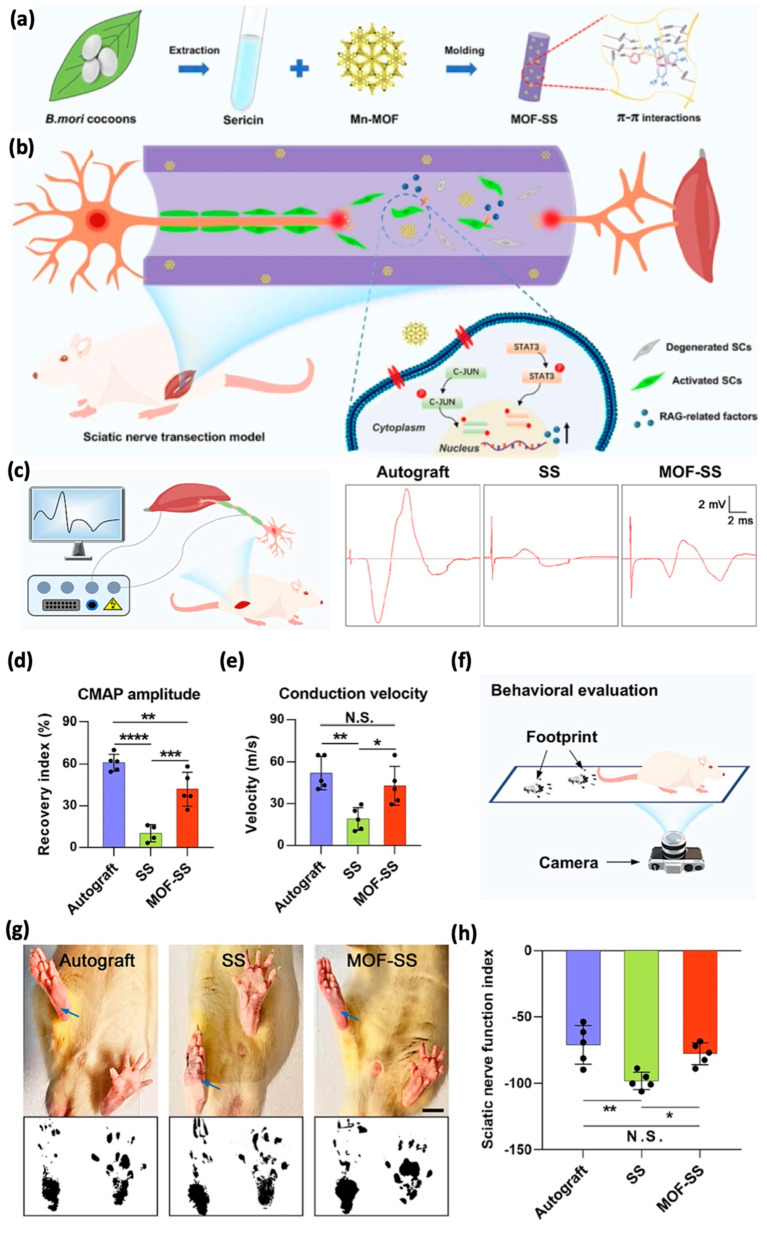
(**a**) Schematic illustration of MOF−SS fabrication and therapeutic mechanism for long-gap PNI nerve regeneration. Mn−MOF is incorporated as a bioactive additive to stimulate RAGs expression in SCs via STAT3 and C-JUN signaling pathways, conferring axon regeneration-promoting properties to MOF−SS. (**b**–**h**) Functional recovery facilitated by MOF-SS. (**b**) Schematics of electrophysiological tests. (**c**–**e**) Representative CMAP patterns (**c**), amplitude quantifications (**d**), and conduction velocity (**e**) for autograft, SS, and MOF−SS groups (* *p* < 0.05; ** *p* < 0.01; *** *p* < 0.001; **** *p* < 0.0001; ANOVA). Behavioral evaluation schematics (**f**), rat hind paw images and footprints with SFI quantifications (**g**,**h**), highlighting improved functional restoration with MOF-SS. Scale bar: 10 mm. Adapted with permission from [52].

Further expanding the sericin scaffold capabilities, manganese/organic frameworks (Mn-MOFs) were introduced into sericin conduits through π-π interactions to fabricate MOF-SS scaffolds (Figure 8) [52]. These novel materials stimulate regeneration-associated genes (RAGs) via both STAT3 and C-JUN signaling pathways. This innovative approach is extremely promising for the treatment of long-gap peripheral nerve injuries, a scenario in which conventional treatments are often ineffective [96]. Indeed, MOF-SS scaffolds support robust axonal regeneration, enhance electrical signal conduction, and reduce muscle atrophy through the reactivation of atrophic Schwann cells and maintenance of a neurotrophic microenvironment. In addition to its regenerative capabilities, sericin has shown promising effects as a cognitive enhancer in Alzheimer’s disease (AD) [97]. Preclinical studies suggest that sericin antioxidant and anti-inflammatory properties may help mitigate neurodegeneration associated with AD by reducing oxidative stress [98], and modulating the ACh levels in the brain cholinergic regions of rats [99]. These neuroprotective effects contribute to the preservation of cognitive functions in animal models, indicating sericin’s potential as a supportive agent in AD therapy. Further investigations are warranted to clarify its mechanisms and optimize delivery strategies for clinical application in neurodegenerative conditions. Sericin’s versatility thus goes beyond scaffold design and includes the possibility of creating multifunctional biomedical devices.

Among the cutting-edge innovations, conductive sericin-derived inks were synthesized by Jeong and Lee, via the grafting of polypyrrole onto methacrylate-modified sericin and poly(ethylene glycol) diacrylate (PEGDA) [100]. All the properties of both biodegradability and electrical conductivity are combined in ultraviolet-crosslinkable inks, enabling their application in neural signal transmission and in bioelectronic interfaces. Apart from exhibiting excellent biocompatibility, the inks are suitable for printing in tailored configurations that may synergize with the body’s intrinsic healing processes and electrostimulation therapies. This double capability affirms materials derived from sericin as novel tools for the treatment of neurodegenerative diseases.

## 3. Challenges and Future Perspectives in Sericin-Based Drug Delivery Systems

The use of sericin as a biopolymer for drug delivery holds immense potential due to its biocompatibility, biodegradability, and multifunctionality, as already described in the previous sections. However, realizing its full potential in pharmaceutical applications requires addressing several critical challenges. A significant challenge is the variability in sericin’s molecular structure, driven by differences in extraction techniques [29,101]. This variability impacts drug encapsulation consistency, release profiles, and therapeutic efficacy. To address these issues, advanced purification technologies, such as size-exclusion chromatography and gradient ultrafiltration, combined with standardized extraction protocols, could ensure sericin’s consistent molecular weight distributions and functional integrity [102,103,104]. Additionally, adopting machine learning-based predictive models could optimize extraction conditions, as has already been performed for silk fibroin, thereby tailoring sericin’s properties for specific drug delivery applications [105].

Another major challenge lies in the physical and chemical stability of sericin-based formulations under environmental stresses, such as pH fluctuations, temperature variations, and ionic changes [106,107,108]. This aspect is particularly critical for chronic disease management, where sustained drug release is necessary. Strategies such as core/shell nanoparticle designs, hybrid materials combining sericin with robust polymers like polylactic acid or polycaprolactone, and nanocrystalline coatings, could enhance stability while retaining biocompatibility. Furthermore, employing crosslinking methods using natural biocompatible agents like genipin or alginate may reduce the reliance on synthetic stabilizers, aligning with the trend toward eco-friendly biomedical innovations [52,109].

Furthermore, while preclinical studies underscore sericin’s safety [110,111,112], regulatory and clinical translations remain bottlenecks. Regulatory frameworks demand rigorous characterization, including immunogenicity, long-term biodegradability, and pharmacokinetics. Although sericin is generally considered biocompatible, its glycoprotein composition raises potential concerns about immunogenicity, particularly in systemic applications. While reported immune responses are typically mild and not clinically severe, [31,32,33], they underscore the need for standardized processing to eliminate residual contaminants and minimize immune activation. Approaches such as enzymatic deglycosylation, advanced purification methods, and surface modifications (e.g., PEGylation) can further reduce immunogenic risk and improve systemic tolerance. To establish sericin’s clinical safety profile, comprehensive in vivo immunogenicity evaluations remain essential. Innovative high-throughput screening technologies could accelerate these evaluations by rapidly assessing the interaction of sericin-based formulations with immune cells and physiological barriers. Additionally, computational simulations could model interactions with biological barriers such as the BBB or gastrointestinal epithelium, paving the way for targeted drug delivery applications in complex diseases like neurodegenerative disorders [113,114].

Sericin’s ability to manipulate apoptotic proteins and mitigate oxidative stress in tumor cells has been well exploited [49,73,74,75]. However, there still seems to be a gap in understanding the cascading pathways involved in subsequent cell disruption and DNA damage. Controlled dose concentrations can help in setting the threshold to avoid toxic effects in healthy cells and more research in death pathways would support exploiting this beneficial property of sericin.

Looking forward, stimuli-responsive systems represent a promising frontier for sericin-based drug delivery. By exploiting sericin’s structural adaptability, researchers can develop platforms that respond to triggers such as pH, temperature, or enzymatic activity. Such systems could enable a localized drug release, significantly improving therapeutic efficacy while minimizing systemic side effects. Moreover, sericin’s amphipathic properties and abundant functional groups provide a foundation for the co-delivery of multiple therapeutic agents. For instance, in cancer therapy, sericin-based carriers could simultaneously deliver chemotherapeutics and immune modulators, fostering synergistic therapeutic effects.

Sustainability is an inherent advantage of sericin, as it is a readily available by-product of the silk industry. Large-scale production using enzyme-assisted extraction methods and energy-efficient processing techniques could reduce the environmental impact and manufacturing costs, making sericin a viable alternative to synthetic polymers [7,115]. Furthermore, integrating sericin production into circular economy frameworks could minimize waste and promote resource efficiency, enhancing its appeal for industrial applications [116,117].

While sericin demonstrates significant promise as a biopolymer for drug delivery, it is useful to compare it with other glycoproteins that have been explored for similar applications (Table 2). Glycoproteins like ovalbumin, mucin, and lactoferrin also offer distinct biological functionalities such as immunomodulation, mucoadhesiveness, or antimicrobial properties, which can influence drug delivery performance [118,119,120,121,122]. For instance, mucin’s mucoadhesive nature makes it highly suitable for transmucosal drug delivery [123,124], while lactoferrin’s ability to bind iron [125,126] and its known antimicrobial properties position it well for targeting infections and inflammatory diseases [127,128,129]. Sericin, by contrast, uniquely combines amphipathic behavior with antioxidant and cytoprotective properties, along with a favorable degradation profile [6,130,131]. Additionally, unlike many glycoproteins that are derived from animal fluids or tissues, sericin is abundantly available as a silk industry by-product, improving sustainability and production scalability [7,132].

A comparative analysis with other biopolymers, such as alginate, chitosan, and hyaluronic acid, highlights sericin’s distinct advantages (Table 3) [107,140,141,142]. Unlike these materials, sericin exhibits inherent bioactivity, including antioxidant, UV-protective, and antimicrobial properties, which add significant value to its role as a drug carrier. However, direct comparisons in areas like long-term safety, targeting specificity, and drug release kinetics remain underexplored. Future studies should prioritize these areas to establish sericin as a superior alternative to existing biomaterials.

Emerging technologies further amplify the potential of sericin-based systems. Bioprinting with sericin-based bioinks could produce patient-specific implants with tailored drug release profiles, advancing precision medicine [143,144]. Similarly, microfluidics offers a pathway to manufacture sericin-based nanoparticles and hydrogels with unparalleled control over size and drug loading efficiency. Smart hybrid systems that combine sericin with conductive polymers, such as polyaniline, could enable dual applications in drug delivery and bioelectronics, such as flexible implantable sensors [145].

Interdisciplinary collaboration is essential to overcome technical and regulatory barriers. Materials scientists, clinicians, and regulatory experts must work together to optimize formulations, streamline approval processes, and design clinical trials. Localized applications like wound healing, where sericin bioactivity and biocompatibility are well-established, could serve as a starting point for clinical validation. Success in these domains would create a roadmap for more complex systemic applications, including targeted therapies for cancer and neurodegenerative diseases. Overall, while challenges such as stability, scalability, and clinical translation remain, the unique properties of sericin position it as a transformative material for drug delivery. By integrating cutting-edge technologies, sustainable practices, and interdisciplinary approaches, sericin-based drug delivery systems could redefine therapeutic paradigms, offering innovative, efficient, and sustainable solutions to pressing medical needs. Sericin offers formulation scientists valuable insights as a multifunctional biopolymer for drug delivery applications. Its physicochemical and bioactive properties enable the development of delivery systems optimized for controlled release, targeted action, and enhanced biocompatibility. A thorough understanding of sericin-based nanocomposites, hydrogels, and scaffolds provides a strong foundation for next-generation pharmaceutical formulations. Moreover, its synergistic interactions with excipients such as silver nanoparticles, chitosan, and other natural polymers support improved formulation stability, efficacy, and safety. Beyond drug delivery, sericin and its role in tissue engineering, regenerative medicine, and wound healing have been widely documented. This has been immensely useful for formulation scientists to scrutinize multidisciplinary platforms for drug development and delivery. With demonstrated potential across antimicrobial, oncological, and neurological therapies, sericin serves as a strategic component in addressing complex drug delivery challenges while meeting regulatory and sustainability goals.

## 4. Conclusions

Sericin-based drug delivery systems have emerged as a potential approach in addressing critical healthcare challenges, including antimicrobial resistance, cancer treatment, and neurodegenerative disease management. Across these diverse applications, sericin’s exceptional properties (biocompatibility, biodegradability, and the ability to form stable, multifunctional carriers) have consistently demonstrated its potential to revolutionize therapeutic strategies. By encapsulating therapeutic agents, providing controlled and targeted release, and enhancing efficacy with its intrinsic bioactive properties, sericin-based systems address longstanding limitations in drug delivery, such as biofilm formation, multidrug resistance, and barriers to effective treatment like the blood/brain barrier in neurological disorders. In antimicrobial therapy, sericin’s versatility has been harnessed in nanoparticles, hydrogels, and nanofibers, offering sustained release, broad-spectrum efficacy, and eco-friendly alternatives to traditional methods. For oncology, its ability to co-deliver drugs, facilitate combination therapies, and integrate advanced modalities like phototherapy and gene silencing has paved the way for safer and more effective cancer treatments. In neurodegenerative disease management, sericin’s role in neural repair scaffolds and drug delivery platforms provides alternatives for tackling diseases that were previously difficult to treat. However, realizing the full potential of sericin-based systems requires overcoming challenges such as scalability, regulatory hurdles, and the need for rigorous clinical validation. Advancements in integrating sericin with emerging technologies offer a path forward to addressing these challenges. The synergy of sericin with compounds such as silver nanoparticles, resveratrol, halloysite nanotubes, and chitosan exhibits a wide range of functionalities and promising biomedical applications. The presented sericin-based nanocomposites and hydrogels show promising outcomes to address key challenges associated with wound care, antimicrobial resistance, and effective drug delivery. Moreover, its environmentally friendly nature aligns with global sustainability goals, further solidifying its position as a material of the future. With continued multidisciplinary research and innovation, sericin stands out to redefine therapeutic criteria across antimicrobial, anticancer, and neurodegenerative applications, offering safer, more effective, and patient-centered solutions to some of the most threatening health issues of this time.

## Figures and Tables

**Table 1:** Overview of sericin-based biomaterials, their fabrication techniques, and biomedical applications.

System Type	Sericin Fabrication/Modification	Key Applications	Examples	References
Nanoparticles	Synthesized via desolvation, self-assembly, or electrostatic interactions	Targeted delivery	Eco-friendly synthesis of sericin/silver nanoparticles (sericin-AgNPs) to enhance antibacterial and wound healing	[38]
	Stabilized with crosslinkers like genipin or glutaraldehyde	Controlled release	Sericin/zeolitic imidazolate framework loaded with doxorubicin as pH-sensitive cancer drug delivery platform	[39]
	Integrated with metallic particles for added functionality	Antimicrobial agents	Sericin-AgNPs-coated nanofibers for bacterial inhibition in food preservation	[40]
Hydrogels	Crosslinked physically, chemically, or enzymatically (e.g., glutaraldehyde, tyrosinase)	Localized delivery	Hydrogel with chitosan and resveratrol as antimicrobial, antioxidant, and wound healing platform	[41]
	Tunable porosity and degradation rates	Tissue engineering	Bilayer scaffold (antimicrobial and pro-angiogenic layers) for enhanced wound healing	[42]
	Combined with bioactive compounds (e.g., lactoferrin, tannic acid)	Immune recovery after chemotherapy	Sericin-based hydrogel dressing with recombinant lactoferrin for chemotherapy-enhanced immune function recovery	[43]
	Temperature-sensitive hydrogels for on-demand drug delivery	Breast cancer treatment	ROS-sensitive tegafur/protoporphyrin IX-loaded injectable hydrogels for combined photodynamic and chemotherapy	[44]
Films	Created via solvent evaporation or casting	Transdermal systems	Sericin/alginate/aloe vera films with hemostatic, antimicrobial, and wound healing properties	[45]
	Combined with chitosan or silk fibroin for enhanced strength	Wound healing and antimicrobial coatings	Sericin-ZnO/AgNPs films for advanced tissue engineering and antibacterial coatings	[46]
	Layer-by-layer assembly to incorporate multiple functionalities	Antimicrobial coatings	Sericin/propolis/fluorouracil films for colorectal cancer treatment	[47]
Micelles and Conjugates	Exploiting sericin amphiphilic properties for micelle formation	High-efficiency drug loading	Antimicrobial and regenerative PDA@Ag/SerMA microneedles for diabetic foot ulcers	[48]
	Environment-responsive triggers like pH	Tumor targeting	Responsive micelles for pH-triggered drug release in tumor microenvironments	[9]
	Combined with photosensitizers for photothermal therapies	Multifunctional cancer therapies	Sericin-poly(γ-benzyl-L-glutamate)-IR780 complexes for photothermal and photodynamic therapies	[49]
Composite Materials	Hybridized with natural/synthetic polymers to enhance specific properties	Multifunctional systems	Sericin/chitosan composites for superior bioactivity in acidic tumor conditions	[50]
	Incorporating metallic nanoparticles (e.g., silver, zinc oxide)	Antimicrobial and wound management	Eri silk-derived sericin-AgNPs scaffolds for multidrug-resistant burn wound treatments	[51]
	Combined with metal/organic frameworks for enhanced regenerative properties	Long-gap peripheral nerve regeneration	MOF-SS scaffolds for nerve repair through activation of regeneration-associated genes	[52]
Nanofibers	Fabricated via electrospinning with functionalized sericin or composites	Antibacterial wound dressings	Sericin-based polyurethane nanofibers with halloysite nanotubes for controlled drug release	[53]
	Integrated with silver nanoparticles	Advanced tissue regeneration and antibacterial system	Sericin nanofiber scaffolds with superior burn wound healing and antimicrobial efficacy	[54]
	Biocompatible nanofibers for sustained drug delivery	Antimicrobial therapies	Sericin/zein nanofibers for stable 5-fluorouracil delivery in cancer treatments	[55]
Smart Carriers	Stimuli-responsive sericin-based materials (e.g., pH, temperature, light-sensitive systems)	Cancer therapy	Sericin charge-reversal nanoparticles for co-delivery of resveratrol and melatonin, exploiting the tumor microenvironment	[56]
	Incorporating therapeutic nanoparticles	Multimodal therapy and diagnostics	Sericin-layered double hydroxides modified with ZnO quantum dots for breast cancer imaging and therapy	[57]
	Combined with superparamagnetic iron oxide nanoparticles (SPIONs)	Gene therapy	Sericin-SPION systems for targeted siRNA delivery in breast cancer treatment	[58]
3D Scaffolds	Engineered using sericin with biomimetic architectures	Skin and tissue regeneration	3D sericin/chitosan/alginate sponges with antibacterial and angiogenic properties for infected wound treatment	[59]
	Incorporating bioactive nanoparticles	Peripheral nerve regeneration	Sericin/manganese/organic framework scaffolds for long-gap nerve injury repair	[52]
	Combining sericin and fibroin in multichannel designs for nerve repair	Neural tissue engineering	Silk fibroin/sericin sponge scaffolds for sciatic nerve regeneration in rat models	[60]

**Table 2:** Comparison of sericin with other glycoproteins used in drug delivery [119,120,121,122,133,134,135,136,137,138,139].

Property	Sericin	Ovalbumin	Mucin	Lactoferrin
Source	Silk cocoon (by-product)	Egg white	Epithelial secretions	Milk, tears, saliva
Molecular Weight Range	10–300 kDa	~45 kDa	200–5000 kDa	~80 kDa
Bioactivity	Antioxidant, antimicrobial, UV-protective	Immunogenic, antigenic	Mucoadhesive, hydrating	Antimicrobial, anti-inflammatory
Biocompatibility	High	Moderate	High	High
DrugEncapsulation	Hydrophilic and hydrophobic drugs	Mostly hydrophilic	Hydrophilic drugs	Hydrophilic drugs
Biodegradability	High	Moderate	Variable	High
Challenges	Variability in extraction, stability	Immunogenicity, denaturation	Viscosity, batch variability	Cost, limited availability
Applications	Controlled release, co-delivery, bioelectronics	Vaccine delivery, antigen carriers	Transmucosal delivery	Infection-targeted therapies, inflammation

**Table 3:** Comparative properties of sericin vs. chitosan, alginate, and hyaluronic acid.

Property	Sericin	Chitosan	Alginate	Hyaluronic Acid
Molecular Weight Range	10–300 kDa	50–2000 kDa	32–250 kDa	10–1000 kDa
Bioactivity	Antioxidant, UV- protective, antimicrobial	Antimicrobial, hemostatic	Biocompatible, gelling agent	Hydrating, anti-inflammatory
DrugEncapsulation	Hydrophilic and hydrophobic drugs	Hydrophilic drugs	Hydrophilic drugs	Hydrophilic drugs
Biodegradability	High	High	Moderate	High
Applications	Stimuli-responsive systems, co-delivery of drugs	Wound healing, drug carriers	Tissue scaffolds, drug carriers	Hydrogels, Targeted delivery

## Data Availability

Not applicable.
